# The Efficacy of Black Chokeberry Fruits against Cardiovascular Diseases

**DOI:** 10.3390/ijms22126541

**Published:** 2021-06-18

**Authors:** Kamila Kasprzak-Drozd, Tomasz Oniszczuk, Jakub Soja, Marek Gancarz, Karolina Wojtunik-Kulesza, Ewa Markut-Miotła, Anna Oniszczuk

**Affiliations:** 1 Department of Inorganic Chemistry, Medical University of Lublin, Chodźki 4a, 20-093 Lublin, Poland; kamilakasprzakdrozd@gmail.com (K.K.-D.); karolina.wojtonik@umlub.pl (K.W.-K.); 2 Department of Thermal Technology and Food Process Engineering, University of Life Sciences in Lublin, Głęboka 31, 20-612 Lublin, Poland; jakubsoja97@wp.pl; 3 Institute of Agrophysics, Polish Academy of Sciences, Doświadczalna 4, 20-290 Lublin, Poland; m.gancarz@ipan.lublin.pl; 4 Department of Lung Diseases & Rheumatology, Medical University of Lublin, 20-093 Lublin, Poland; ewa.markut-miotla@umlub.pl

**Keywords:** *Aronia melanocarpa* L., black chokeberry, polyphenols, metabolic syndrome, metabolic diseases, type 2 diabetes, obesity, cardiovascular diseases

## Abstract

Epidemiological studies have emphasized the association between a diet rich in fruits and vegetables and a lower frequency of occurrence of inflammatory-related disorders. Black chokeberry (*Aronia melanocarpa* L.) is a valuable source of biologically active compounds that have been widely investigated for their role in health promotion and cardiovascular disease prevention. Many in vitro and in vivo studies have demonstrated that consumption of these fruits is associated with significant improvements in hypertension, LDL oxidation, lipid peroxidation, total plasma antioxidant capacity and dyslipidemia. The mechanisms for these beneficial effects include upregulation of endothelial nitric oxide synthase, decreased oxidative stress, and inhibition of inflammatory gene expression. Collected findings support the recommendation of such berries as an essential fruit group in a heart-healthy diet. The aim of this review was to summarize the reports on the impact of black chokeberry fruits and extracts against several cardiovascular diseases, e.g., hyperlipidemia, hypercholesterolemia, hypertension, as well as to provide an analysis of the antioxidant and anti-inflammatory effect of these fruits in the abovementioned disorders.

## 1. Introduction

In these times of more and more common civilization diseases, to a large extent attributable to an unhealthy lifestyle marked by stress, environmental pollution and improper nutrition, people are looking for food products that replace synthetic ingredients with natural counterparts [1,2]. It is known that some foods can offer other beneficial effects on top of supplying the most essential nutrients. There is increasing scientific evidences to support the notion of proof of the presence of a remarkable association between a diet rich in fruits and vegetables and a lower incidence of different chronic pathologies, such as cancers, infections, obesity, neurodegenerative and cardiovascular diseases. In these, a chronic inflammatory state is the major contributing factor to their development [3,4]. Berries, and, among them, black chokeberry (*Aronia melanocarpa* L.) are fruits immensely rich in in natural compounds, including vitamins, minerals, dietary fibers and polyphenols [5,6,7].

In the nineteenth century, *A. melanocarpa* was brought to Europe from the eastern part of North America, where it grows wild in the form of deciduous shrubs, reaching even 2–3 m in height. Initially, it aroused the interest of the Russian botanical gardens, from where it spread to Central and Eastern Europe. The shape of the fruit resembles a small apple (they are round or slightly elongated). The berry is 6–13 mm in diameter and weighs 0.5–2 g. *Aronia* has low soil nutrition requirements and is resistant to pests. In various European countries, black chokeberry is commonly used to produce juices, jams, jellies, teas, wines and tinctures. It is also a source of natural food dyes. Due to the tart taste and bitter almond smell of the raw fruit, chokeberry is used in concoction with other fruits, e.g., apples, pears or black currants, in the production of fruit juices or teas.

These preserves are gaining more and more recognition among consumers, due to fact that black chokeberry is a good source of polyphenols [8], especially anthocyanins [9]. In the last years, these compounds have attracted considerable attention because of their recognized antioxidant activity and potential in health promotion and disease prevention [10,11,12]. In epidemiological and clinical studies, aronia and its polyphenolic constituents have been associated with improved cardiovascular risk profiles. Consumption of these fruits is associated with significant improvements in hypertension, LDL oxidation, lipid peroxidation, total plasma antioxidant capacity, dyslipidemia and glucose metabolism. There are reports that mechanisms for these beneficial effects are related to include upregulation of endothelial nitric oxide synthase, decreased oxidative stress and inhibition of inflammatory gene expression [5,13,14].

The aim of this review was to summarize the reports on the impact of black chokeberry fruits and extracts against the cardiovascular diseases e.g., hyperlipidemia, hypercholesterolemia, hypertension, as well as to provide an analysis of the antioxidant and anti-inflammatory effect of these fruits in above mentioned disorders.

## 2. Main Bioactive Compounds of Chokeberry

### 2.1. Polyphenolic Compounds—Introduction

Polyphenolic compounds are biofactors that determine the high-level bioactivity of chokeberry fruits. Black chokeberries are some of the most substantial sources of such chemical compounds [15,16,17,18]. Phenolic are natural compounds that have an aromatic ring with at least one hydroxyl group. Their structure can vary from simple compounds, to complex polymers with high molecular weight. According to the most common classification, polyphenols are normally subdivided into two main groups: flavonoids (i.a. anthocyjanins, flavonols, flavanols, flavanones, and isoflavones) and non-flavonoids (i.a. phenolic acids, xanthones, stilbens, lignans, and tannins) [18].

### 2.2. Flavonoids

**Figure 1:**
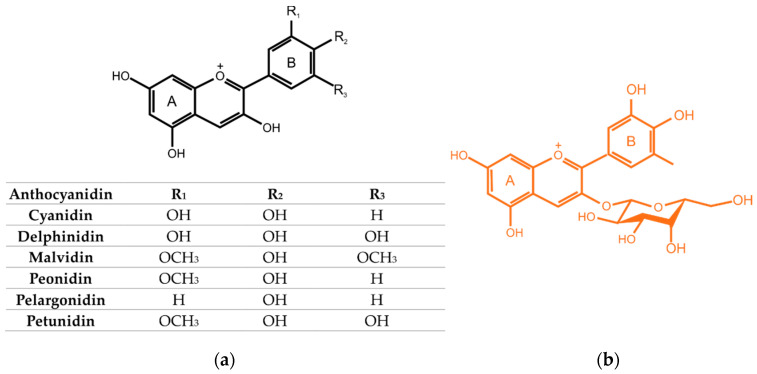
Chemical structure of most widespread anthocyanidins (**a**) and cyanidin 3-O-galactoside (**b**).

Flavonoids with the C_6_–C_3_–C_6_ backbone (i.e., 2-phenylchromen-4-one) are the most abundant and widely distributed plant metabolic polyphenols [19,20]. Anthocyanins are a group of flavonoid, polyphenol chemical compounds. Their characteristic feature is the fact that they give color to fruits and flowers. This varies from red-orange to blue-violet [17,[20]]. They are soluble in water, and their schematic structure includes aglycone (anthocyanidin), sugar(s) and, very often, acyl group(s) [21]. The majority of the identified anthocyanins are based on the six most widespread anthocyanidins. Their chemical structure is shown in Figure 1a [21]. According to Denev at al. [22], the total indicated amount of anthocyanins in fresh chokeberries varies from 357 to 1790 mg/100 g fresh weight (FW). Oszmiański and Wojdylo [23] stated that anthocyanins constitute 25% of the content of all polyphenols. Other researchers (Jakobek et al. [24]) believe this value to be 41%. The anthocyanin composition is represented almost exclusively by cyanidin glycosides [25]. Cyanidin 3-O-galactoside is the predominant anthocyanin found in chokeberry fruit and its content is about 65% of the anthocyanin total (Figure 1b) [21]. Other anthocyanins, the contents of which in chokeberries are significant are cyanidin-3-arabinose, cyanidin-3-glucoside and cyanidin-3-xyloside [26]. In addition, there are small amounts of pelargonidine-3-galactoside and pelargonidine-3-arabinoside in the relevant fruits [27,28].

Phenolic compounds are unstable plant secondary metabolites. Their stability is considerably affected by environmental factors, such as light, presence of copigments, self-association, metallic ions, enzymes, oxygen, ascorbic acid, sugar [29]. Anthocyanins are extensively degraded by thermal treatment, and their concentration declines linearly [30]. It was investigated that storage at 70 °C for 24 h leads to an approximately 50% decrease in the total anthocyanin content in freeze-dried chokeberry fruit powder [21].

Chokeberry fruits contain a certain amount of flavanols (proanthocyanidins—polymers and oligomers composed of flavan-3-ol units, especially epicatechin) and flavonols (mainly represented by quercetin glycosides) [25,31]. The fruit pulp contains the majority of procyanidins (about 70%), while the peel holds 25%, and the seeds—about 5% [32]. Flavonols represent merely 1.3% of the total chokeberry phenolics [23]. Among others, the identified quercetin derivatives include 3-O-(6′-O-β-arabinosyl-ß-glucoside), 3-O-(6′-α-rhamnosyl-β-galactoside), 3-O-(6′-α-rhamnosyl-β-glucoside), 3-O-β-galactoside and 3-O-β-glucoside. Their content is estimated to be about 71 mg/100 g FW [33]. Furthermore, in the fruits of *A. melanocarpa* also contain insignificant amounts of quercetin 3-vicianoside [25].

### 2.3. Phenolic Acids

Phenolic acids are compounds composed of an aromatic ring, carboxyl groups, hydroxyl and/or methoxy groups. Depending on the number of carbon atoms in the molecule, phenolic acids can be classified into two basic groups: benzoic and cinnamic acid derivatives. Fruits are characterized mostly by the presence of free phenolic acids [18]. 

**Figure 2:**
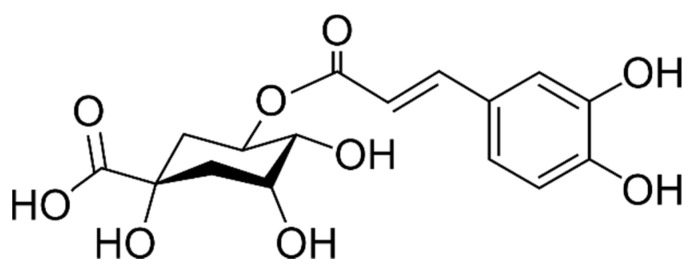
Chemical structure of chlorogenic acid.

The total content of phenolic acids of *A. melanocarpa* fruits has been published to be 63.9 [34], 96 [35], 121.9 [36] mg/100 g FW. There is a consensus of opinion that the most abundant is chlorogenic acid, which is a complex of caffeic acid linked to quinic acid through an ester bond. Its chemical structure is shown in Figure 2. This compound together with neochlorogenic acid are considered to be the major non-flavonoid polyphenols in chokeberries [25,31]. It has been suggested that chlorogenic acid is a natural copigment present in fruits, and comes about through the copigmentation process [33].

According to Häkkinen et al. [37], significant amounts of caffeic, ferulic and *p-*coumaric acids have been detected in chokeberry samples. *p-*hydroxybenzoic acid has also been determined, but in small amounts [37]. The other indicated phenolic acids are cryptochlorogenic acid, derivatives of *p-*coumaric acid, caffeic acid and its derivatives, protocatechuic, vanillic, ferulic, salicylic, syringic and ellagic acids. The juice also contains esters of chlorogenic and neochlorogenic acids, as well as phenylacetic acid derivatives [15]. Chokeberry phenolic acids are unstable, especially hydroxycinnamic acid, which decreases in quantity during juice pasteurization (80 °C) by up to 59% [38].

### 2.4. Bioavailability of Chokeberry Polyphenols

Bioavailability is one of the main factors affecting the efficacy of bioactive substances with regard to the human body. Because bioactive substances ingested by the body are transformed or degraded during the process of gastrointestinal digestion, ingested bioactive substances cannot be fully utilized by the body [39]. The bioavailability of phenolic compounds is affected by interaction with other macromolecules such as proteins, carbohydrates and lipids [40,41]. The metabolism of polyphenolic compounds are related to their bioavailability—absorption, transport, distribution and retention in the biological fluids, cells and tissues. Overall, the level of bioavailability of chokeberry phenolic compounds is quite low [17,22,25]. 

The low bioavailability of anthocyanins is caused, inter alia, by the fact that a small fraction of these compounds is made available through use in the human stomach, while most are utilized by the small intestine or metabolized by the intestinal microflora [39]. Lower medium pH values bring about greater anthocyanin stability [42]. In animal models (rats), after absorption, anthocyanins are mostly distributed to the urinary bladder and kidney [43]. As a type of conjugation, glucuronidation and methylation are the major pathways in chokeberry anthocyanin metabolism [17]. According to Kay et al. [44], cyanidin-3-glycosides are rapidly absorbed and metabolized extensively following a moderate-to-high oral dose in humans. The results obtained by Yu et al. [39] indicate that after simulated gastrointestinal digestion, the total phenol content and anthocyanin content of an intestinal digestion group decrease by 53.64% and 70.45%, respectively, within 2 h. Thus, aronia anthocyanins are recovered in blood and urine in nanomolar concentrations [22]. In addition, proanthocyanidins are narrowly absorbed—predominantly in the lower intestine [22].

Ingestion of free forms of hydroxycinnamic acids results in their rapid absorption in the stomach and small intestine, followed by conjugation through detoxification enzymes. Because the dietary concentration of hydroxybenzoic acids is much lower than hydroxycinnamic acids, there are limited experiences about their bioavailability studies [45]. Chlorogenic and neochlorogenic acids (mainly contained in chokeberry fruits) are hydroxycinnamic acid derivatives and they are naturally esterified. This impairs their absorption. Human biological fluids and tissues largely lack esterases capable of hydrolyzing chlorogenic acid to release caffeic acid, which shows higher antioxidant activity [22]. The major part of chlorogenic acid, therefore, escapes absorption in the small intestine and reaches the colon, this site playing a pivotal position in metabolism and absorption [46,47]. The role of bifidobacteria (*B. animalis*) in the hydrolysis of chlorogenic acid by gut microbiota [47] to quinic and caffeic acid has been noted [46].

Polyphenols are very sensitive to pH heat and light. These compounds can be quick-ly oxidized with a considerable loss in activity. Therefore, the development of novel for-mulation methods to stabilize and protect polyphenols from degradation and, conse-quently, to improve their bioactivity attracts considerable interest. The utilization of en-capsulated polyphenols instead of free compounds can overcome the drawbacks of their instability, alleviate unpleasant tastes or flavors, as well as improve the bioavailability and half-life of the compound [48].

Encapsulation is a technology for packing small solid particles, liquid droplets, or gas molecules in a form that can release the contents at controlled rates under specific conditions [49]. By this method, substances are enclosed within a layer of coating or embedded in a homogeneous or heterogeneous matrix obtaining particles such as microcapsules or microspheres according to their internal structure, core-shell-like or matrix, respectively. The size of microparticles can range from 1 micron to a few millimeters, but in most cases is less than 200 microns. Particles characterized by a smaller size—from 1 nanometer to 1 micrometer there are nanoparticles [50]. The coating materials may include natural or synthetic polymers and lipids. In general, three steps are involved in the encapsulation of bioactive agents: the formation of the wall around the material to be encapsulated, ensuring that undesired leakage does not occur and ensuring that undesired materials are kept out. For polyphenol many encapsulation methods are used in order to protect the core material from environmental factors (light, pH, temperature, moisture, and oxygen), while improving the shelf stability of the final product and the sustained delivery of the encapsulate. Techniques applied to this group of natural substances can be classified in three main categories: physical (spray-drying and encapsulation processes using supercritical fluids), physico-chemical (coacervation, ionic gelation, emulsions, and methods based on hydrophobic interaction: micelles, liposomes), and chemical approaches (is in situ polymerization) [51].

## 3. Antioxidant Activity of Chokeberry

Most commonly available literature reports suggest that oxidative stress is important in the pathogenesis of various disorders and diseases of civilization, therefore the attention of the general public has been drawn to the role of antioxidants in their prevention and treatment [52]. Antioxidants are low molecular weight chemical compounds, the common feature of which is the ability to significantly delay or prevent the oxidation of other substances [53,54]. The term also takes into account the fact that antioxidant concentrations are relatively low in relation to molecules subjected to oxidation reactions [55]. According to the more specific definition proposed by Apak et al. [56], antioxidants are substances of natural or synthetic origin that prevent or inhibit oxidative cell damage caused by the action of physiological oxidants and which show a clear positive reduction potential. This definition emphasizes the important role of antioxidants in the proper functioning of the body at the cellular level and their potential influence on the maintenance of health [53].

Chokeberry fruits have high antioxidant potential, usually higher than other analyzed plant materials. The antioxidant activity of chokeberries was confirmed in various radical scavenging assays (i.e., the use DPPH, ABTS), the effects of transition metals on changes in the state of oxidation (FRAP), and the ability to inhibit lipid peroxidation in a variety of model systems [15,22].

Aronia juice exhibits the highest antioxidant capacity among the polyphenol-rich beverages. Trolox equivalence antioxidant capacity (TEAC) for aronia juice is four times higher than that of cranberry, blueberry juice or red vine [33]. Kähkönen et al. [57] analyzed 92 different phenol-rich berry extracts. Herein, the most considerable antioxidant activity and the highest total phenolic content were found in chokeberry and crowberry extracts. Studies conducted by Zheng and Wang [58] also recognized the dominate antioxidant activity of aronia in comparison to other common berries (e.g., blueberries, cranberries). This highest antioxidant activity result has been confirmed, inter alia, in the research of Tolić et al. [59] In this, an ORAC assay was followed by a scavenge superoxide radicals evaluation. In this case, elderberries and blueberries were found to be more active than chokeberry fruits. The results of this test are 5783, 5646, 5165 µmol TE/g FW (micromole Trolox equivalents per gram of dry extract), respectively. In the described study, chokeberry extract was found to be the most potent inhibitor of lipid peroxidation and had the highest TRAP value of 4051 μmol TE/g [60].

It has been noted that chokeberry extract, measured by the DPPH radical dot method, demonstrates higher radical scavenging activity on being compared to that of butylated hydroxyanisole (BHA) and butylated hydroxytoluene (BHT). These last are used in the food industry as synthetic antioxidants [61]. In this work, antioxidant and free-radical scavenging properties of extracts of the dried chokeberry pomace from different years (2016 and 2017) were evaluated using DPPH and electron paramagnetic resonance (EPR) spectroscopy (DPPH-EPR). The resulting figures were 6417 (year 2016) and 11,127 µmol TE/g (year 2017). It was thus concluded that the content of phenolic compounds (including anthocyanins) in chokeberry fruits is higher in the years with hot and dry weather. Summer 2016 in northeastern Poland (harvest place) was warmer than that of 2017 [31].

There is positive correlation between the total polyphenol content (TPC) and the antioxidant activity of aronia samples [22]. Large differences in the content of phenolic compounds depending on the variety of chokeberry, as well as climatic conditions during ripening and harvesting have been indicated. The exemplary TPC values obtained and published for *A. melanocarpa* fruits are: 40.06 mg GAE (gallic acid)/g [62], 106,337.20 mg GAE/kg [24], 690–2560 [25], 1079–1921 [63], 3955.28–4393.50 [64] mg GAE/100 g FW [25], 10,386–14,350 mg/kg FW [24].

The uppermost antioxidant activity of chokeberry (compared to other fruits) is believed to relate to its significant content of anthocyanins and phenolic compounds and depends on their structure. Phenolics such as quercetin and cyanidin, with 3′,4′-dihydroxy substituents in the B ring and conjugation between the A and B rings, had highly effective radical scavenging structures in the berries. Some polyphenolic acids display high antioxidant activity, as well, probably because of their ability to be hydrogen donors due to dihydroxylation in the 3,4 positions [25,58].

In the recent study of Denev et al. [17], minor phenolic components, such as quercetin and epicatechin demonstrated the highest ORAC and TRAP antioxidant activity. Given the amount of individual phenolics in the fruits, proanthocyanidins are the major contributor to the antioxidant activity of fresh black chokeberries. However, the researched polyphenols and preparations had no effect on the spontaneous chemiluminescence (CL) of human neutrophils, and only a mild effect on PMA (phorbol-myristate-acetate)-activated CL [17]. It has been noted that cyanidin-3-galactoside purified from chokeberries is capable of inhibiting lipid peroxidation induced either by Fe(II) ions, UV irradiation, or 2,2-azobis (2-amidinopropane) dihydrochloride (AAPH) peroxyl radicals, in a liposomal membrane system [65]. An experimental study was conducted in which the parameters of oxidative stress were assessed in twenty male Wistar rats, incl. by determining the concentration of substances reacting with thiobarbituric acid (TBARS) and the activity of glutathione peroxidase (GSH-Px). For 3 months, 10 rats received only clean water to drink, the remaining rats received an additional 10–100 mg/L of chokeberry anthocyanins. Here, the administered anthocyanins significantly reduced the content of TBARS and thiol protein groups and non-significantly increased glutathione peroxidase activity, total content of antioxidants and nitrite concentration. These observations led to the conclusion that chokeberry anthocyanins decrease lipid peroxidation and can potentially be used to combat oxidative stress [66]. Of note, it has been shown that in vitro cranberry fruit extract significantly reduces oxidative stress (secondary to chemotherapy and surgery) in platelets in patients with invasive breast cancer [67].

Another experiment investigated the effect of increased consumption of anthocyanins contained in chokeberry juice on the indicators of oxidative stress in rowers during a training camp. The competitors were divided into 2 groups: one daily receiving 150 mL of aronia juice containing 23 mg of anthocyanins/100 mL and a control. Before and after a month of supplementation, an exercise test was performed. Blood samples were taken before the exercise test, one minute after the test, and after a 24-h rest. In the supplemented group, TBARS concentrations were statistically significantly lower than in the control group. This group also had lower GSH-Px and superoxide dismutase activity, which was an observation until then unconfirmed by the results of other studies. These differences may result from the different observation times and population variability (including physical performance). Over all, the results suggest that increased anthocyanin intake reduces exercise-induced oxidative damage to red blood cells, most likely by enhancing the endogenous antioxidant defense system [68]. Kardum et al. [69] investigated a group of 25 healthy women who consumed aronia juice. (100 mL a day, for three months). The juice increased the superoxide dismutase and glutathione peroxidase activity. The proof of protection against oxidation was the increase in polyunsaturated fatty acid (PUFA) share in erythrocyte membranes.

## 4. Anti-Inflammatory Effect of Chokeberry Fruits

Oxidative stress causes excessive production of reactive oxygen species (ROS) in the cells and tissues when the antioxidant system cannot neutralize them. Imbalance in this protective mechanism can lead to the damage of cellular molecules such as DNA, proteins and lipids. Reactive oxygen species and/or reactive nitrogen species (RNS) overproduction, for example, can cause adverse reactions in cellular structures and functions. Various stimuli such as excessive ROS/RNS production, endotoxins, viruses, fatty acids, cellular redox status, cytokines, growth factors and carcinogens and some natural or artificial factors and substances may, therefore, initiate the inflammatory process [70].

**Figure 3:**
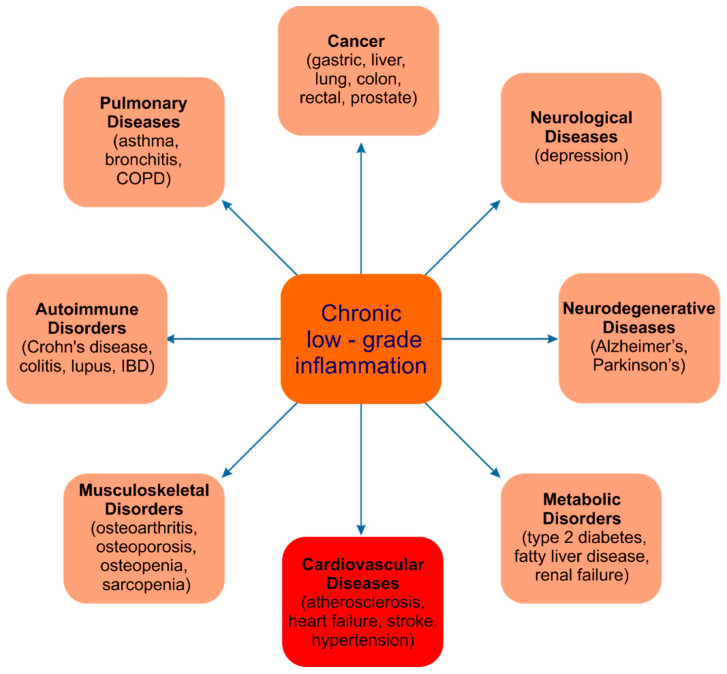
Diseases associated with chronic low-grade inflammation.

Under physiological conditions, inflammation is the protective and temporary response of the immune system to unfavorable stimuli. Inflammatory responses are characterized by the production of cytokines, which act as signals between immune cells. These include interleukin (IL)-1β, IL-6 and tumor necrosis factor-α (TNF-α), as well as IL-10. The central orchestrator of the inflammatory response is the nuclear factor kappa-light-chain-enhancer of activated B cells (NF-kB), a redox-sensitive transcription factor [4]. Other important mediators of inflammation include pattern recognition receptors such as Toll-like receptors (TLR) and kinases such as mitogen-activated protein kinase (MAPK) [71]. Significantly, most cardiovascular diseases are accompanied by chronic low-grade inflammation (meta-inflammation, Figure 3) [72].

In research, aronia fruit polyphenols were found to reduce the development of many cardiovascular diseases by increasing immune defenses and reducing inflammation. Currently, black chokeberry is highlighted particularly in relation to strengthening the human immune system. This effect occurs with the participation of different mechanisms of action, such as inhibition of release of cytokine IL-6, IL-8 and TNF-α in the human monocytes and activation of NF-κB and prostaglandin E2 (PGE2) [25]. Experiments conducted by Ho et al. [73] in mice have demonstrated that procyanidins and anthocyanins are mainly responsible for the immunomodulation effect of these fruits.

Appel et al. [74] examined the role of aronia juice concentrate in lipopolysaccharide-treated human primary monocytes isolated from peripheral blood and RAW264.7 macrophages. The results indicated that chokeberry significantly inhibited the release of TNF-α, IL-6 and IL-8 in monocytes and the activation of the NF-κB pathway in macrophages. Similar findings were obtained in lipopolysaccharide-treated BV2 cells and in mice, where chokeberry ethanolic extract significantly reduced tissue damage in the hippocampus by downregulating cyclooxygenase 2 (COX-2), inducible nitric oxide synthase (iNOS) and TNF-α levels [75]. Increased expression of TNFα plays an important role in the enhancement of apop-totic processes in cardiomyocytes, endothelial cells and the development of myocardium structural-functional alterations. Analysis of the caspase-3 activity in cardiomyoblasts from myocardium tissue of rat embryos preincubated with aronia extract and treated with TNFα showed the anti-apoptotic activity of this preparation [76]. Above researches emphasize protective and anti-inflammatory role of chokeberry fruits.

Administration of chokeberry fruit extract for patients after myocardial infarction treated with statins causes changes in the value of inflammatory markers (decrease in the concentration of IL-6, intercellular adhesion molecule (ICAM), vascular cell adhesion molecule (VCAM), C-reactive protein (CRP) and monocyte chemoattractant protein-1 (MCP1) and increase in adiponectin level) [77]. Qin and Anderson [78] studied the levels of body inflammation markers in rats with metabolic syndrome induced by a high fructose diet. They administered to animals aronia fruit extract and observed reduction in TNFα and IL-6 levels and a significant increase in adiponectin concentration. Decreases in the concentrations of TNFα and IL-6 cytokines after supplementation with aronia extract were also observed in the blood plasma of rats with artificially induced hypertension [79]. Other studies revealed that in patients with metabolic syndrome, however, a slightly increased CRP level was not significantly reduced after two months of black chokeberry intake [13].

In a study by Zapolska-Downnar and Bryk, aronia extract demonstrated an anti-inflammatory effect and inhibited TNFα stimulated transcription of the ICAM-1 and VCAM-1 genes, thus lowering the expression of adhesion molecules in human aortic endothelial cells (HAECs) in experimental studies. Furthermore, the adhesion of peripheral blood mononuclear leukocytes to the endothelium of the aorta was reduced. Moreover, this extract lessened the activation of the nuclear transcription factor under the influence of TNFα and decreased the production of ROS in HAECs. The inhibition of adhesion molecules was related to the activity of anti-inflammatory drugs (e.g., dithiocarbamate, ibuprofen, pyrrolidine) [80]. This effect may result from the presence of anthocyanins (cyanidin, delphinidin and peonidin derivatives) in chokeberry fruits. These compounds prevent the adhesion of monocytes to TNFα-activated human umbilical vein endothelial cells (HUVECs), the initial step in atherosclerosis development, under physiologically-relevant conditions [81].

In other research, chokeberry extract reduced the level of monocytes and granulocytes responsible for inflammation in diabetic rats and increased the concentration of lymphocytes inhibiting the formation of atherosclerotic plaques [82]. These results confirm that aronia is a fruit potentially useful in the prevention and treatment of cardiovascular diseases connected with low-grade inflammation.

## 5. Role of Chokeberry in Hyperlipidemia and Hypercholesterolemia

Research indicates that a diet rich in *A. melanocarpa* berries and their preserves improved the lipid balance of people with metabolic syndrome. These fruit decreased levels of triacylglycerols (TAG), total cholesterol (TC) and low-density lipoprotein (LDL) in the blood [83] (Table 1).

In studies conducted by Nowak et al. [84] (Table 1), aronia juice reduced high TAG level in women. In other research, *A. melanocarpa* juice taken with glucomannan lessened HDL levels in obese women [85] (Table 1).

These activities result from the antioxidant capacity and anthocyanin content in black chokeberry products [86,87,88,89,90]. However, the antioxidant capacity of various fresh berries and its products are often different. Fresh aronia fruits, extracts and juice contain the most effective combination of biologically active compounds [33]. However, while the research conducted by Kulling and Rawel indicated that aronia extract reduces high levels of TAG, TC, and LDL in hypertensive rats and improves the level of HDL lipoprotein [91], that of Daskalova et al. demonstrated that the juice changes only the LDL concentration [89] (Table 1). Moreover, Lipińska and Jóźwik [87] (Table 1), in feeding merino lambs with chokeberry pomace (300 g of pomace/kg of feed) found that the feed increases the HDL and decreases the TAG level, however, the TC and LDL concentrations remain unchanged. Other authors [92] conducted research on inactivated apolipoprotein E (ApoE) in mice which were supplemented with chokeberry extract. They observed that after four weeks of feeding the mice, their plasma TC levels decrease, but no differences in plasma TAG levels were found. In addition, there were no significant changes in the expression of genes responsible for the mechanisms of cholesterol metabolism, lipogenesis and lipid β-oxidation.

Non-alcoholic fatty liver disease (NAFLD) is a disorder associated with lipid metabolism. It is a type of fatty liver that does not result from excessive alcohol consumption. Risk factors for this type of fatty liver include: metabolic syndrome, obesity, type 2 diabetes, hypertension, and insulin resistance [93]. Lipogenesis was significantly reduced in mice with NAFLD, however, that followed a diet with a high proportion of fat (41% of energy), cholesterol (1.5 g/kg) and sucrose (340 g/kg) and freeze-dried black chokeberry powder. Here, lipogenesis was limited by the effect of the powder. Moreover, TAG was markedly reduced. The TAG decrease was accompanied by changes in the expression of selected mRNAs associated with de novo lipogenesis and TG levels in hepatocytes (acetyl-CoA carboxylase, sterol regulatory element binding protein 1 and fatty acid synthase) [88] (Table 1). Yamane et al. [90] (Table 1) fed male mice a diet with 10% of lyophilised black chokeberry fruit. At 28 days after starting a normal diet, a high fat diet and a high-fat diet containing 10% freeze-dried aronia berries, triglyceride, total cholesterol and LDL cholesterol levels were measured. In this study, was found that mild fibrosis induced by a high-fat diet was reduced in livers of mice fed a high-fat diet containing aronia berries. Results of test showed that total lipids weight, serum triacylglycerol, liver and serum LDL, fatty acid binding protein 1, and fatty acid binding protein 4 were reduced in mice fed a high-fat diet containing aronia berries.

While aronia is a source of numerous bioactive phenolic compounds, one group deserves special attention. These are the anthocyanins, which are powerful antioxidants that show significant anti-adipogenic properties [94]. Park et al. [95] observed that chokeberry powder dissolved in water (at a dose of 50 mg/kg/day) improved the hepatic lipid metabolism of mice fed a high-fat diet. This was manifested by the prevention of lipid accumulation and the reduction of liver weight gain. The same experiment revealed that consumption of aronia decreases the levels of fatty acid synthase, leptin, triacylglycerols, mRNA expression and PPARγ2 factor that are involved in hepatic lipid metabolism. PPARγ2 is a protein that regulates transcription of the target genes of adipocyte protein 2 binding free fatty acids (FFA), and lipoprotein lipase (LPL). This study showed that the aronia powder affects the metabolism of the hepatic lipids by way of the PPARγ2-dependent pathway. Moreover, due to FFA, mouse hepatocytes increase the expression of genes involved in lipid metabolism and lipid accumulation. In addition, black chokeberry fruit extract (concentrations of 40 and 80 μg/mL) was found to decrease the expression of mRNA PPARγ2, and P2 and LPL in hepatocytes and limit the accumulation of lipids by 7% and 33.4%, respectively.

Kim et al. [92] investigated the effect of polyphenol-rich black chokeberry extract on the expression of genes involved in intestinal lipid metabolism. In the experimental studies, caco-2 cells were incubated with black chokeberry extract (50 or 100 μg/mL for 24 h) for quantitative real time polymerase chain reaction analysis. The researchers found that the extract limits the expression of genes for cholesterol synthesis, uptake and efflux in a dose-dependent manner. This study demonstrated that expression of genes for cholesterol synthesis (3-hydroxy-3-methylglutaryl coenzyme A reductase and sterol regulatory element binding protein 2), apical cholesterol uptake (Niemann-Pick C1 Like 1 and scavenger receptor class B Type 1) and basolateral cholesterol efflux (ATP-binding cassette transporter A1) decreases under the influence of black chokeberry extract. In the work, significant increase in the levels of LDL receptors and cellular LDL uptake was also observed. This implies that cholesterol was taken into the cell, broken down and used in further processes (membranę synthesis, bile and steroid production). Abnormalities in haemostasis are factors that can contribute to the formation of atherosclerosis. They may cause thrombosis, which limits blood flow to the organs. Anthocyanins from black chokeberry can be regarded as inhibitors of the LDL oxidation that is the key mechanism of atherosclerosis. Broncel et al. [94] revealed that aronia extract anthocyanins (three times for 100 mg of extract per day for 3 months) had influence upon blood pressure, endothelin, serum lipids, lipid peroxidation and oxidative status of persons with metabolic syndrome. After the therapy in patients was observed, a significant decrease of total cholesterol, LDL fraction, triglycerides, enzyme catalase and systolic blood pressure (SBP) was indicated. Other human studies showed that chokeberry extract supplementation of patients with hypercholesterolemia decreased their high cholesterol level and lipid peroxidation in erythrocytes. The study showed that the consumption of the extract improved the rheological properties of erythrocytes [86] (Table 1).

## 6. Impact of Chokeberry on Platelet Function

In the work of Bijak et al., extracts of black chokeberry presented strong anticoagulant activity by prolonging blood-clotting times and decreasing the maximal velocity of fibrin polymerization in human plasma. These findings confirmed the in vitro anticoagulant properties of aronia [96]. Research conducted by Olas et al. [97] has shown that aronia extract reduces platelets adhesion to collagen and platelet aggregation, as well as ROS production in blood platelets (in resting plates and in platelets activated by thrombin). Thus, chokeberry fruits can prevent thrombosis in pathological states where plasma procoagulant activity and oxidative stress are observed.

In other studies [98,99], the effect of chokeberry extract on the clot formation (in vitro) and the fibrin lysis during the model of hyperhomocysteinemia was investigated. Here, a high level of homocysteine in the blood plasma was found to contribute to increased risk of atherosclerosis and thrombosis. Furthermore, homocysteine and homocysteine thiolactone (its most reactive form) stimulated fibrinogen polymerisation, reduced fibrin lysis, and increased oxidative stress in the plasma. Their work indicated that inhibition of the superoxide anion formation by aronia extract is a possible mechanism for platelet activation reduction and stimulation of thrombin adhesion to collagen and fibrinogen in a hyperhomocysteinemia model.

Results of studies conducted by Ryszawa et al. [100] revealed the impact of aronia extract on platelet function (in vitro and in vivo) in subjects with significant cardiovascular risk factors (diabetes mellitus, smoking, hypertension and hypercholesterolemia). However, in vivo research confirmed that the extract induces a decrease in superoxide production only in patients with cardiovascular risk factors. This effect was not observed in a group without risk factors for arteriosclerosis. Still, the extract demonstrated anti-aggregatory effects on platelets in both studied groups.

Olas et al. [97] studied the antioxidant properties of aronia extract on oxidative/nitrative stress (in vitro) in human blood platelets. They noted that the extract significantly inhibits platelet protein carbonylation and thiol oxidation. Furthermore they put forward that quercetin glycosides, anthocyanidins and phenolic acids contained in aronia in particular may contribute to the prevention of peroxynitrite-related cardiovascular diseases. Bijak et al. [96] evaluated the effect of black chokeberry extract against nitrative and oxidative damage induced by peroxynitrite. They estimated that the extract significantly inhibits both nitration of the fibrinogen molecule and the formation of high molecular weight protein aggregates.

Sikora et al. [101] examined the complex mechanism of the influence of black chokeberry polyphenols on platelet activity *(*in vitro studies). Their work demonstrated that aronia polyphenols brings about inhibition of the amidolytic activity of the main fibrinolytic enzyme (plasmin) and enhances the contribution of other plasma components and fibrinogen in the modulation of haemostasis.

## 7. Influence of Chokeberry on Hypertension

The renin-angiotensin-aldosterone system (RAAS) plays an important role in the regulation of blood pressure and fluid and electrolyte balance. Angiotensin I is formed from angiotensinogen by renin (released by granular cells in the arterial wall that supplies blood to the glomerulus). It is broken down into angiotensin II by way of the angiotensin converting enzyme (ACE). Angotensin II has a very strong vasoconstrictive effect, and stimulates the secretion of aldosterone by the cells of the adrenal cortex. In addition, ACE inactivates bradykinin, a potent vasodilatory peptide [102].

Sikora et al. [13] demonstrated that *A. melanocarpa* fruit extract reduce significantly the activity of ACE. The aim of study was to analyze the effects of two-month supplementation with aronia preparation on the activity of ACE in patients with metabolic syndrome (MS). Their findings revealed that ACE activity decreased by 25% (range: 8–53%) after the first month and by 30% (range: 4–80%) after two months. This study confirmed previous observations that the effects of chokeberry extract on ACE are the strongest in patients with higher activity of this enzyme and with higher levels of arterial pressure [77]. The comparison of the efficacy of the ACE inhibition in the blood by captopril and aronia preparations also indicated that the potency of the antihypertensive effect of the chokeberry results from multiple mechanisms of action (not only from the ACE inhibition) [13].

In studies conducted in spontaneously hypertensive rats a diet with 10% of lyophilised chokeberry reduced their blood pressure after three days [103] (Table 2). However, a statistically significant reduction of the SBP occurred after 28 days. Here, ACE activity was found to decrease in the kidneys, remain unchanged in the serum, and increase in the lungs. What is more, lyophilised *A. melanocarpa* juice and extract applied to SHR rats at a dose of 50 mg/kg for ten days significantly reduced their SBP and DBP (diastolic blood pressure). Here, the strongest reduction in blood pressure occurred 3 h after the administration. However, the changes in the pressure were periodic, not so high (for DBP) and did not increase on the days following the start of the study (with regard to SBP). The study concluded that the hypotensive effect of chokeberry preserves was not only induced by the phenolic compounds, but also by other components that may act independently or in synergy with polyphenols.

Besides being responsible for increases in blood pressure, Angiotensin II exerts a deleterious effect on human endothelial progenitor cells (EPC). These determine proper functioning of the cardiovascular system. In research, aronia extract reduced the harmful effect of Ang II on the EPC functions. Herein, it increased EPC proliferation, decreased senescence of cells, improved functionality, adhesion, angiogenic potential and migration capacity [104].

Nitric oxide (NO) plays an important role in regulating blood pressure. Hellström et al. [108] found using spontaneously hypertensive rats, that aronia polyphenols may affect NO production by activating endothelial nitric oxidase enzyme (independently of ACE). Varela et al. [109] reached similar conclusions. During their study, chokeberry extract activated the endothelial nitric oxide synthase (eNOS) in bovine coronary artery endothelial cells. Of interest, the concentration of eNOS was strongly positive correlated with the concentration of NO produced. The highest degree of eNOS activation was obtained after 10 min of administration of the extract.

With regard to aronia administration, apart from the inhibition of the ACE activity and the increase of NO production, other mechanisms of action reducing blood pressure include changes in the concentration of the hormone endothelin-1 (ET-1) and improvements in antioxidative properties. The administration of three doses of 100 mg of *A. melanocarpa* extract daily for two months was found to decrease ET-1 in subjects with metabolic syndrome. At the same time, the changes in the SBP and DPB were normalised [94] researcher concluded that the hypotensive effect was due to the antioxidative effect of the extract on the status of the organism. Ciocoiu et al. noticed increased total antioxidant capacity, an increase in the number of enzymes that provide endogenous defence against glutathione peroxidase and superoxide dismutase, as well as enhanced concentration of endogenous glutathione [79] (Table 2). Apart from the aforementioned, the chokeberry extract was found to inhibit histopathological changes and normalise cardiac and renal tissue structures [91,107].

Sikora et al. [110] conducted a study on patients treated with aronia fruit extract (3 × 100 mg/day) for two months. As a result, the patient SBP, LDL lipoprotein and cholesterol decreased. In other research, Kardum et al. [85] administered chokeberry supplement (chokeberry juice and glucomannan) to 20 obese women aged 45–65 (daily volume 100 mL for one month). This preparation decreased the systolic blood pressure from 127.6 to 116.4 mmHg (on average). In another study, chokeberry juice was administered for four weeks (daily dose 200 mL) to adults (33–67 years) who were not pharmacologically treated for high-normal blood pressure or grade I hypertension. Here, administration of the juice significantly decreased patients 24-h pressure and the patients pressure of the non-sleeping time [69,106] (Table 2). Finally, Loo et al., [105] (Table 2) served for patients with hypertension 300 mL of aronia juice and 3 g of aronia powder daily for eight weeks. In consequence, the DBP measured every 15 min from 7.00 a.m.–9.59 p.m. was significantly reduced.

**Figure 4:**
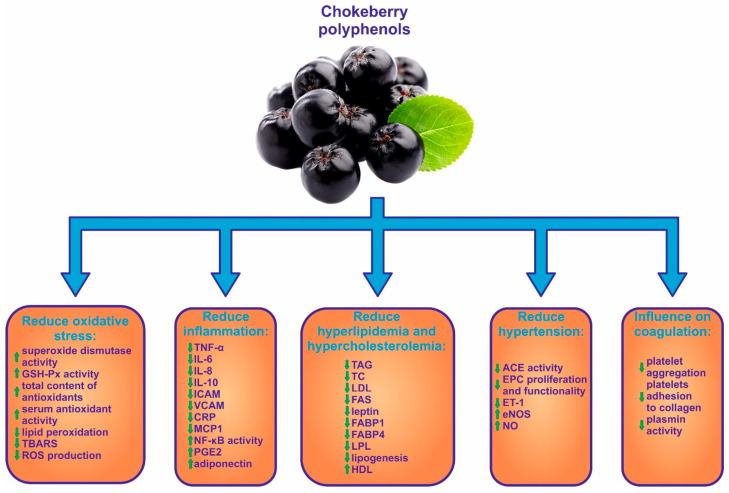
Role of chokeberry fruits in cardiovascular dysfunction.↑value increase; ↓value decrease; ACE: angiotensin converting enzyme, CRP: C-reactive protein, eNOS: endothelial nitric oxide synthase, EPC: endothelial progenitor cells, ET-1: endothelin-1, FABP1: fatty acid binding protein 1, FABP4: fatty acid binding protein 4, FAS: faty acid synthase, GSH-Px: glutathione peroxidase, HDL: high-density lipoprotein cholesterol, ICAM: intercellular adhesion molecule, IL-6: interleukun 6, IL-8: interleukun 8, IL-10: interleukun 10, LDL: low-density lipoprotein cholesterol, LPL: lipoprotein lipase, MCP1: monocyte chemoattractant protein-1, NF-κB: nuclear factor kappa-light-chain-enhancer of activated B cells, NO: nitric oxide, PGE2: prostaglandin E2, ROS: reactive oxygen species, TAG: triacylglycerol, TBARS: substances reacting with thiobarbituric acid, TC: total cholesterol, TNF-α: tumor necrosis factor-α, VCAM: vascular cell adhesion molecule.

The results of research conducted by Zhao et al. [14] confirmed that the protective role of fruits (including black chokeberry) against cardiovascular disease involves a protective vascular endothelial function, modulation of blood pressure, regulation of lipid metabolism, reduction of oxidative stress, attenuation of inflammation, inhibition of platelet function and suppression of thrombosis (Figure 4).

## 8. Conclusions

Black chokeberry possesses a high amount of nutritive and biologically active compounds such as polyphenols, with flavonoids and anthocyanins being most representative. Numerous studies have highlighted the efficacy of the berry and its extracts or constituents against chronic low-grade inflammation and in diseases resulting from it. Recent researches indicate numerous positive effects of consumption of *A. melanocarpa* products. Their health-promoting activity was especially noted in cardiovascular diseases e.g., hypertension, hyperlipidemia and hypercholesterolemia. The therapeutic effect of aronia also includes its impact on the homeostasis of patients with metabolic syndrome. The presented review summarized the numerous benefits resulting from chokeberry intake and its inclusion in a daily diet.

## Figures and Tables

**Table 1:** Selected human and animal studies concerning effects of black chokeberry on lipid and cholesterol level.

Black Chokeberry form and Dose	Study Model	Mechanism of Actions/Effects	Ref.
Alcoholic extract of chokeberry fruits (10%), 30 mL a day for 40 days	Human study: patients with hypercholesterolemia, arterial hypertension and deregulated protein metabolism	TC ↓, LDL ↓, TAG ↓, glucose ↓	[83]
Organic chokeberry juice; 250 mL a day for 3 weeks	Human study: healthy, non-smoking subjects with correct BMI	TAG ↓, serum antioxidant capacity (DPPH) ↑,	[84]
Chokeberry supplement prepared from pure juice enriched with 2 g of stable glucomannan fibers; 100 mL a day for 4 weeks	Human study: postmenopausal women with abdominal obesity	BMI ↓, WC ↓, SBP ↓, HDL ↓, GSH-Px ↑; membrane fatty acid profile in erythrocytes: MUFA ↓, 18:1n-9 ↓, n-3 PUFA ↑, 22:6n-3 ↑, n-6/n-3 ↓, unsaturation index ↑	[85]
Chokeberry extract; 100 mg 3 times a day for 2 months	Human study: patients with hypercholesterolemia without pharmacological treatment, healthy individuals as a control group.	erythrocytes: TC ↓, lipid peroxidation ↓, parameter S ↓; beneficial influence on rheological properties of erythrocytes	[86]
Chokeberry pomace; diets with the addition of 150/300 g of chokeberry pomace per each kg of the feed mixture.	Animal study: Polish Merino lambs	ALT ↓, AST ↓, CK ↓, GGT ↓, LDH ↓, TAG ↓, glucose ↓, HDL ↑	[87]
Dried chokeberry powder; high fat diet with 0.5% or 1% chokeberry powder for 8 weeks.	Animal study: C57BL/6 J mice with NAFLD induced by high cholesterol, high fat and sucrose diet	TAG ↓, liver weight ↓, abdominal fat ↓, SREBP-1 ↓, ACC ↓, FAS ↓,	[88]
Sterilized chokeberry juice; 25 mL a day for 3 months	Animal study: male Wistar rats	BW ↑, BMI ↑, LDL ↓, retarded age-related changes in the aortic wall.	[89]
Freeze-dried chokeberry fruits; diet containing 10% freeze-dried fruits for 28 days.	Animal study: C57BL/6JmsSlc male mice fed a high-fat diet	reduced liver fibrosis; total lipids weight ↓; liver: TAG ↓, TC ↑, LDL ↓; serum: TG ↓, LDL ↓; FABP1 ↓, FABP4 ↓.	[90]

↑ value increase; ↓ value decrease; ACC: acetyl-CoA carboxylase, ALT: alanine transaminase, AST: aspartate transaminase, BMI: body mass index, BW: body weight, CK: creatine kinase, FABP1: fatty acid binding protein 1, FABP4: fatty acid binding protein 4, FAS: fatty acid synthase, GGT: gamma-glutamyl transferase, GSH: glutathione, GSH-Px: glutathione peroxidase, HDL: high-density lipoprotein cholesterol, LDH: lactate dehydrogenase, LDL: low-density lipoprotein cholesterol, MUFA: monounsaturated fatty acid, PUFA: polyunsaturated fatty acid, SREBP-1: sterol regulatory element binding protein 1, TC: total cholesterol, TAG: triacylglycerol, WC: waist circumference.

**Table 2:** Selected human and animal studies concerning effects of black chokeberry on hypertension.

Black Chokeberry form and Dose	Study Model	Mechanism of Actions/Effects	Ref.
Cold-pressed chokeberry juice and convection oven dried chokeberry powder; 300 mL juice and 3 g powder a day for 8 weeks	Human study: subjects with mildly elevated blood pressure, no regular use of antihypertensive drugs	day DBP ↓, dU-potassium ↓; inflammation markers: IL10 ↓, TNFα ↓,	[105]
Organic chokeberry juice; 100 mL a day for 12 weeks	Human study: healthy women	24 h SBP ↓, parameters oxidative status: TBARS ↓, PAB ↓, TAC ↓, DZOase ↑	[69]
Organic chokeberry juice; 200 mL a day for 4 weeks	Human study: subjects with presence of high normal BP or grade I hypertension, no regular use of antihypertensive drugs	TAG ↓, 24 h SBP ↓, 24 h DBP ↓, awake SBP ↓, awake DBP ↓	[106]
Freeze-dried chokeberry fruits; normal diet containing 10% chokeberry fruits for 28 days.	Animal study: SHR rats	SBP ↓, lung ACE ↑, kidney ACE ↓	[103]
Powdered black chokeberry ethanol extract; 0.050 g/kg every 2 days, for 8 weeks.	Animal study: wistar white rats with induced arterial hypertension	GSH-Px ↑, GSH ↑, TAC ↑, SBP ↓, DBP ↓	[107]

↑ value increase; ↓ value decrease; ACE: angiotensin I-converting enzyme, DBP: diastolic blood pressure, dU: 24-h urinary excretion, GSH: glutathione, GSH-Px: glutathione peroxidase, IL: interleukin, LDH: lactate dehydrogenase, MUFA: monounsaturated fatty acid, PAB: pro-oxidant-antioxidant balance, SBP: systolic blood pressure, TAC: total antioxidant capacity, TBARS: thiobarbituric acid-reactive substances, TNF-α: tumour necrosis factor α, TAG: triacylglycerol.
